# Supplementary material for: A region-resolved mucosa proteome of the human stomach
Source: Nat Commun. 2019 Jan 3;10:39. doi: 10.1038/s41467-018-07960-x (PMC6318339; doi:10.1038/s41467-018-07960-x)
Supplement: Supplementary file 1 — Supplementary Information [file 41467_2018_7960_MOESM1_ESM.pdf]

A Region-resolved Mucosa Proteome of the Human Stomach

Ni et al.

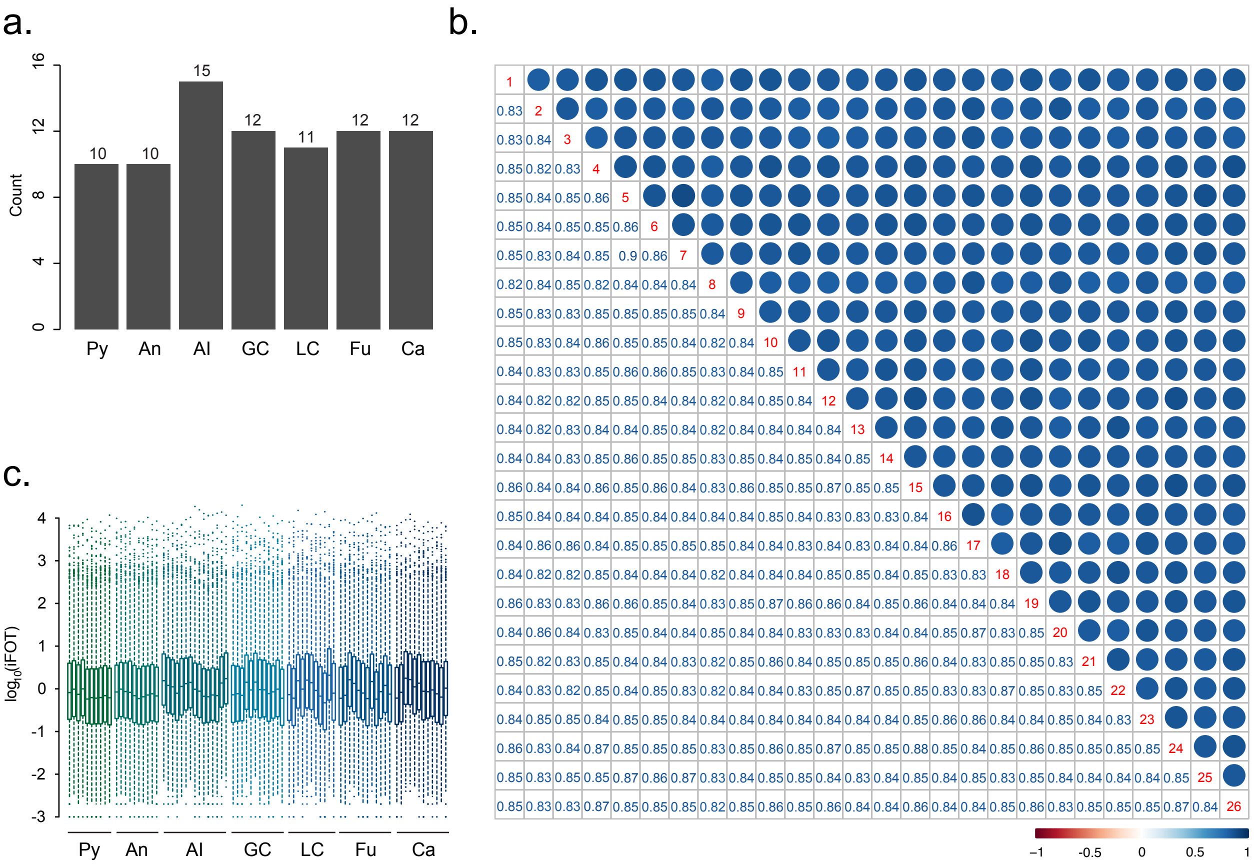

Supplementary Figure 1. Number of mucosa tissue samples, MS reproducibility, and the proteomes of 82 mucosa samples.

a) Number of mucosa samples collected from the 7 anatomic stomach regions. **b)** Correlation of 26 quality control samples. **c)** Intensity distributions of 82 normal mucosa samples across the 7 anatomic stomach regions (center line: median, bounds of box: 25th and 75th percentiles, and whiskers: from Q1-1.5*IQR to Q3+1.5*IQR).

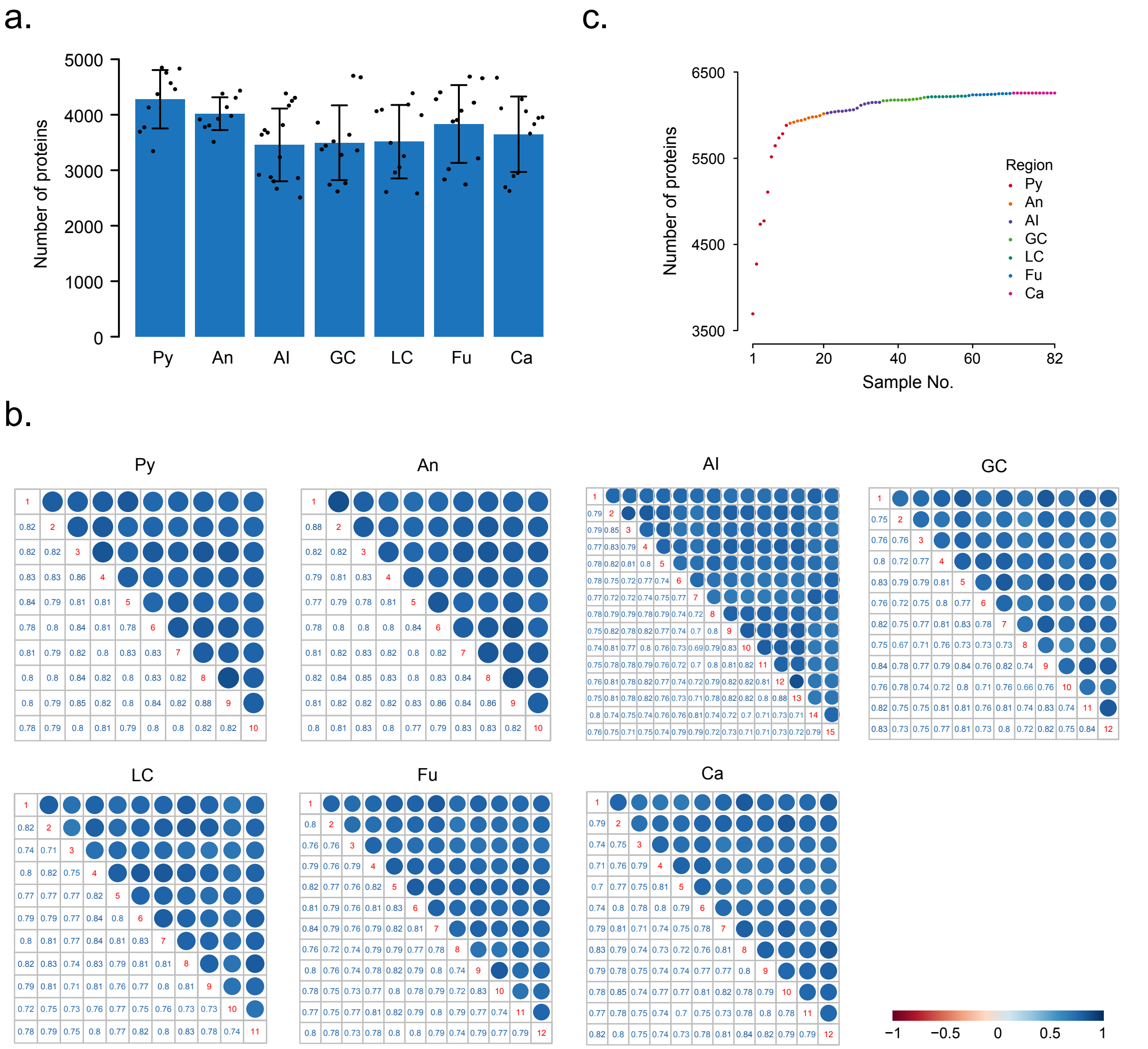

Supplementary Figure 2. The mucosa proteome coverages, intra-region sample correlations, the cumulative number of gene products.
a) Bar chart indicating the average number of the identified proteins from the 7 region (Py: n=10, An: n=10, AI: n=15, GC: n=12, LC: n=11, Fu: n=12, Ca: n=12). Mean ± SD. **b)** Spearman correlations within patient from the 7 regions. **c)** The cumulative number of gene products after filtering across the 7 regions.

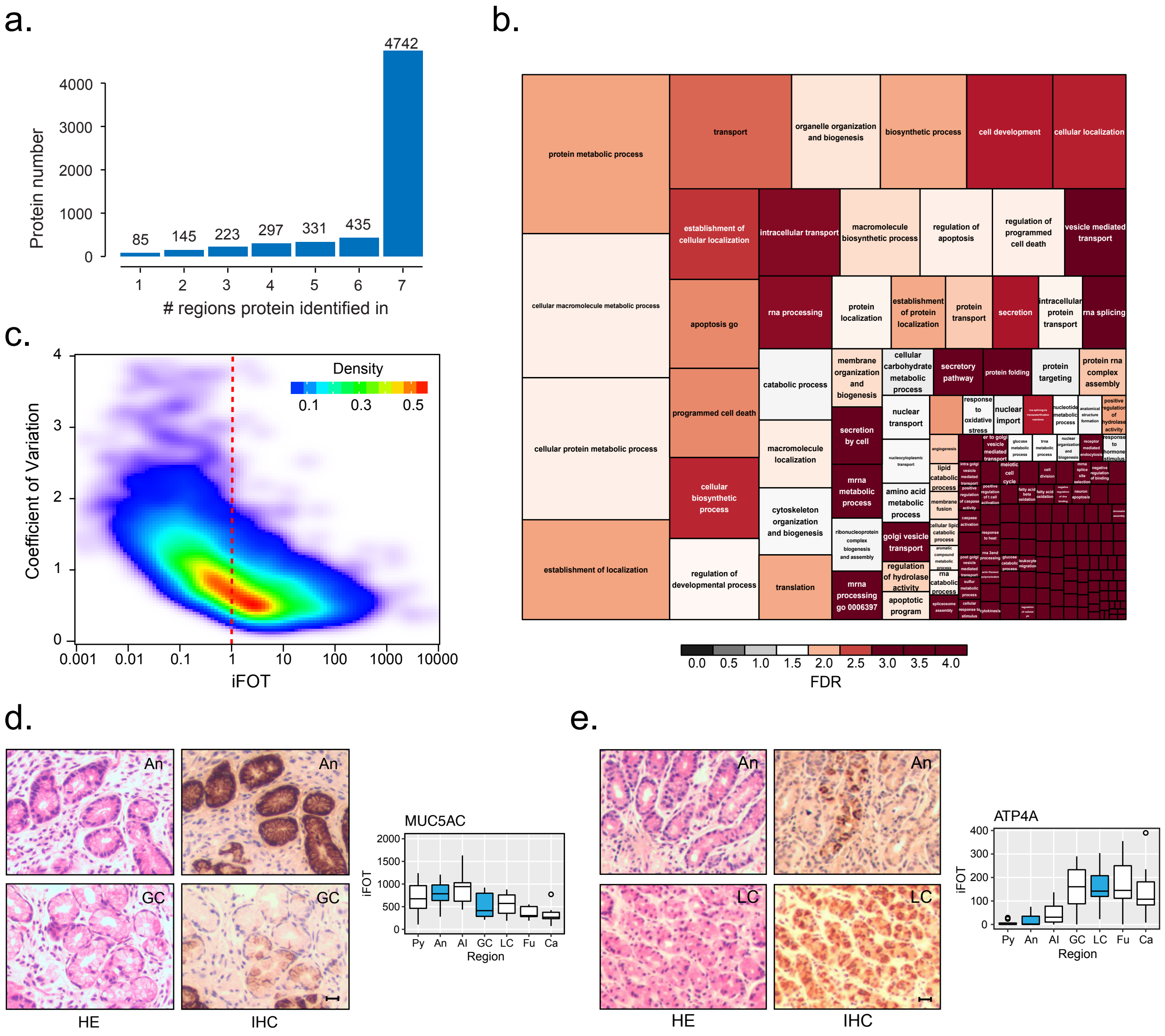

Supplementary Figure 3. Mucosa core proteome and their functions, protein abundances, and HE & IHC of MUC5AC and ATP4A.

a) Bar chart indicating the number of proteins expressed in 1-7 regions. Most proteins were expressed in all the 7 regions. **b)** Gene Ontology treemap for the 4,742 core proteins. The box size is proportional to the number of genes appeared in the GO term. **c)** Distributions of protein abundances and coefficient of variations (CVs). **d)** Hematoxylin-eosin (HE) and immunohistochemistry (IHC) staining of MUC5AC in the region An and GC. Scale bar: 100 μm. The boxplot displaying the iFOT intensity of MUC5AC in the 7 regions (center line: median, bounds of box: 25th and 75th percentiles, and whiskers: from Q1-1.5*IQR to Q3+1.5*IQR). **e)** Hematoxylin-eosin (HE) and immunohistochemistry (IHC) staining of ATP4A in the region An and LC. Scale bar: 100 μm. The boxplot displaying the iFOT intensity of ATP4A in the 7 regions (center line: median, bounds of box: 25th and 75th percentiles, and whiskers: from Q1-1.5*IQR to Q3+1.5*IQR).

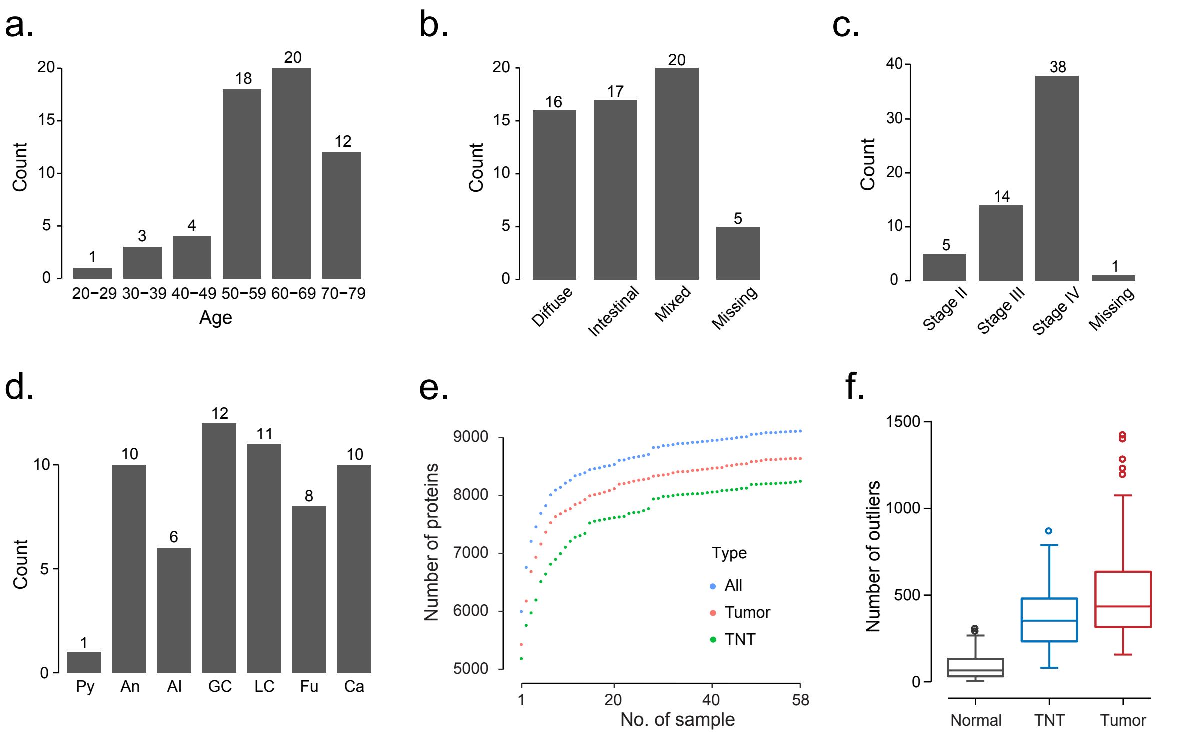

Supplementary Figure 4. Mucosa proteomes from 58 gastric cancer patients.
a) Distribution of ages of gastric cancer patients. **b)** Distribution of histological subtypes of gastric cancer. **c)** Distribution of stages of gastric cancer. **d)** Number of GCA mucosa samples collected in the 7 regions. **e)** The cumulative number of gene products identified in tumors, tumor nearby tissues, and all gastric cancer tissues. **f)** The number of outlier proteins in normal samples, tumor nearby tissues, and tumors (center line: median, bounds of box: 25th and 75th percentiles, and whiskers: from Q1-1.5*IQR to Q3+1.5*IQR).

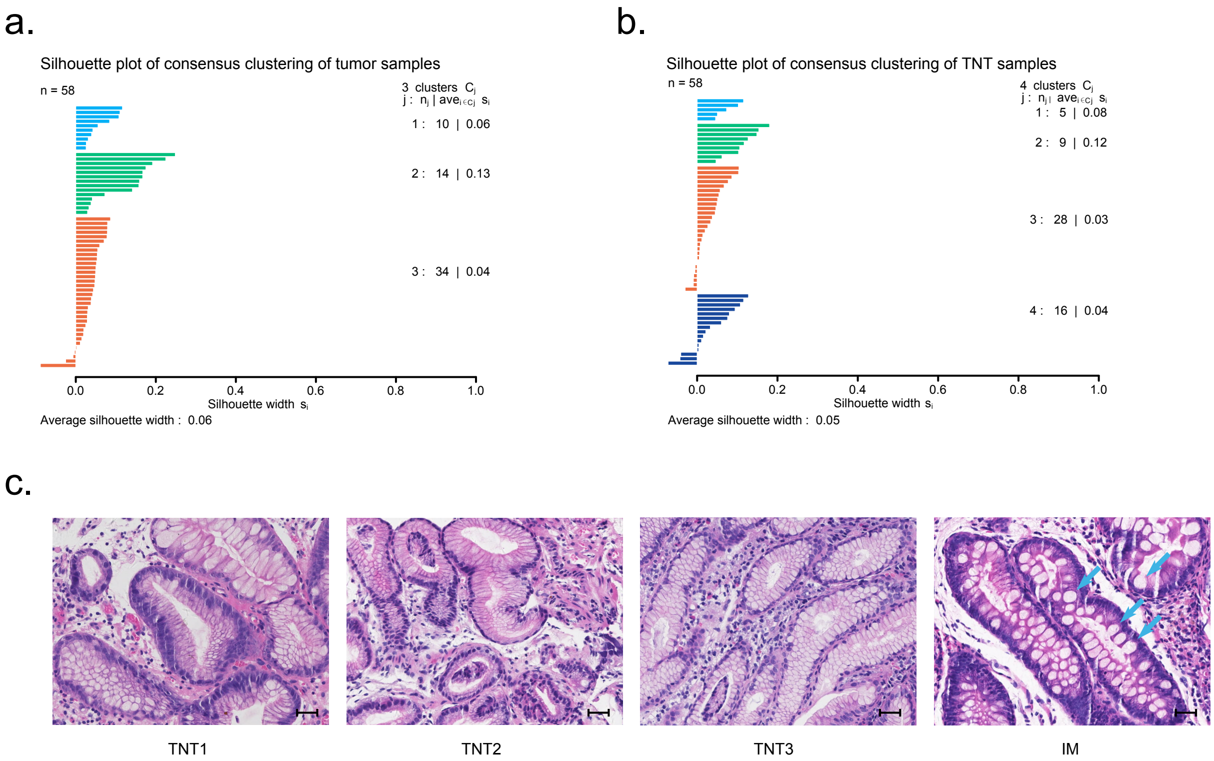

Supplementary Figure 5. Gastric cancer subtyping.
a) Silhouette plot of consensus clustering of tumor samples. **b)** Silhouette plot of consensus clustering of tumor nearby samples. **c)** Hematoxylin-eosin (HE) staining of three I/C protein specific cancer type TNT samples and one intestinal metaplasia (IM) sample. Blue arrows: goblet cells. Scale bar: 100 μm.
