# Supplementary material for: A region-resolved mucosa proteome of the human stomach
Source: Nat Commun. 2019 Jan 3;10:39. doi: 10.1038/s41467-018-07960-x (PMC6318339; doi:10.1038/s41467-018-07960-x)
Supplement: Supplementary file 3 — Description of Additional Supplementary Files [file 41467_2018_7960_MOESM3_ESM.docx]

**Description of Additional Supplementary Files**

**File Name:** Supplementary Data 1

**Description:** 82 mucosa tissue samples with detailed clinical information.

**File Name:** Supplementary Data 2

**Description:** Data information of the 82 normal mucosa samples. Sheet 1 A list of 13,401 gene products and their relative abundances (iFOT) in 82 normal mucosa samples. Sheet 2 A list of 6,258 gene products and their relative abundances (iFOT) in 82 normal mucosa samples after filtering (Relate to Fig. 1b). Sheet 3 The non-normalized data of 82 normal samples and the quality measure information. Sheet 4 The detection frequency of the 6,258 gene products in the 7 regions. Sheet 5 Pylorus (Py) and antrum (An) enriched proteins. Sheet 6 Gene Ontology terms of the Py/An enriched proteins.

**File Name**: Supplementary Data 3

**Description:** Core proteome and section specific proteins of 82 normal mucosa samples. Sheet 1 Gene Ontology terms of the core proteome calculated by ClueGO in Cytoscape. Sheet 2 Student’s t-test, ratio and the expression distribution of 6,258 gene products listed in Supplementary Table 2-shee 2.

**File Name:** Supplementary Data 4

**Description:** 5 protein modules by co-expression analysis and their biological functions. Sheet 1 Lists of gene products enriched in 1-5 gene modules Sheet 2-6 Biological functions of gene products enriched in 1-5 gene modules

**File Name:** Supplementary Data 5

**Description:** 58 gastric cancer patients and their clinical information.

**File Name:** Supplementary Data 6

**Description:** Data information of the 58 pair of tumor(T)-tumor nearby tissue(TNT) mucosa samples. Sheet 1 A list of 15,579 gene products and their relative abundances in 58 pair of T-TNT mucosa samples. Sheet 2 A list of 9,110 gene products and their relative abundances (iFOT) in 58 pair of T-TNT mucosa samples after filtering (Relate to Fig. 1b). Sheet 3 Outlier proteins of the 116 gastric cancer samples. Sheet 4 The non-normalized data of 58 pair of T-TNT mucosa samples and the quality measure information.

**File Name:** Supplementary Data 7

**Description:** GC subtypes clustered by the T or TNT samples. Sheet 1 The subtype of the T or TNT samples. Sheet 2 The iFOT distribution of the T and TNT samples; the detected frequency and the outlier ratio of the proteins in T and TNT samples; the subtype specificity of the proteins. Sheet 3-5 Biological functions of 3 subtypes (T1-3) based on tumor tissues. Sheet 6-8 Biological functions of 4 subtypes (TNT 1-4) based on tumor nearby tissues.

**File Name:** Supplementary Data 8

**Description:** A list of stomach/intestine specific genes from the Human Protein Atlas database.
