# Supplementary material for: A region-resolved mucosa proteome of the human stomach
Source: Nat Commun. 2019 Jan 3;10:39. doi: 10.1038/s41467-018-07960-x (PMC6318339; doi:10.1038/s41467-018-07960-x)
Supplement: Supplementary file 12 — Reporting Summary [file 41467_2018_7960_MOESM12_ESM.pdf]

nature research

Corresponding author(s): Jianming Xu, Bei Zhen and Jun Qin

Reporting Summary

Nature Research wishes to improve the reproducibility of the work that we publish. This form provides structure for consistency and transparency in reporting. For further information on Nature Research policies, see Authors & Referees and the Editorial Policy Checklist.

Statistical parameters

When statistical analyses are reported, confirm that the following items are present in the relevant location (e.g. figure legend, table legend, main text, or Methods section).

n/a	Confirmed	
☐	☒	The exact sample size (*n*) for each experimental group/condition, given as a discrete number and unit of measurement
☐	☒	An indication of whether measurements were taken from distinct samples or whether the same sample was measured repeatedly
☐	☒	The statistical test(s) used AND whether they are one- or two-sided<br>*Only common tests should be described solely by name; describe more complex techniques in the Methods section.*
☒	☐	A description of all covariates tested
☒	☐	A description of any assumptions or corrections, such as tests of normality and adjustment for multiple comparisons
☐	☒	A full description of the statistics including central tendency (e.g. means) or other basic estimates (e.g. regression coefficient) AND variation (e.g. standard deviation) or associated estimates of uncertainty (e.g. confidence intervals)
☐	☒	For null hypothesis testing, the test statistic (e.g. *F*, *t*, *r*) with confidence intervals, effect sizes, degrees of freedom and *P* value noted<br>*Give P values as exact values whenever suitable.*
☒	☐	For Bayesian analysis, information on the choice of priors and Markov chain Monte Carlo settings
☒	☐	For hierarchical and complex designs, identification of the appropriate level for tests and full reporting of outcomes
☐	☒	Estimates of effect sizes (e.g. Cohen's *d*, Pearson's *r*), indicating how they were calculated
☐	☒	Clearly defined error bars<br>*State explicitly what error bars represent (e.g. SD, SE, CI)*

Our web collection on statistics for biologists may be useful.

Software and code

Policy information about availability of computer code

Data collection	MS raw files were processed with the Firmiana proteomics workstation. Raw files were searched against the NCBI human Refseq protein database (released on 04-07-2013, 32,015 entries) in Mascot search engine (version 2.3, Matrix Science Inc).
Data analysis	The region specific proteomes acquired by the proteome analysis of 7 regions in stomach was visualized by a Circular proteome map generated with the software Circos (version 0.67-7). The functionally organized GO term network of 4,742 core proteins was calculated by ClueGO (version 2.5.2) in the software Cytoscape (version 3.6.1). Correlation analysis was performed by using the corrplot package in R software (version 0.84). Hierarchical clustering analysis and principal component analysis (PCA) were implemented in R software. Go term enrichment analysis of proteins was based annotations in the Gene Set Enrichment Analysis (GSEA) database (v5.2).

For manuscripts utilizing custom algorithms or software that are central to the research but not yet described in published literature, software must be made available to editors/reviewers upon request. We strongly encourage code deposition in a community repository (e.g. GitHub). See the Nature Research guidelines for submitting code & software for further information.

Data

Policy information about availability of data

All manuscripts must include a data availability statement. This statement should provide the following information, where applicable:

- Accession codes, unique identifiers, or web links for publicly available datasets
- A list of figures that have associated raw data
- A description of any restrictions on data availability

MS raw files and searching output data are deposited into proteomeXchange via the iProX partner repository with the accession number PXD011821.

Field-specific reporting

Please select the best fit for your research. If you are not sure, read the appropriate sections before making your selection.

☒ Life sciences ☐ Behavioural & social sciences ☐ Ecological, evolutionary & environmental sciences

For a reference copy of the document with all sections, see nature.com/authors/policies/ReportingSummary-flat.pdf

Life sciences study design

All studies must disclose on these points even when the disclosure is negative.

Sample size	The 82 apparently normal mucosa samples were obtained from 36 living individuals by endoscopic stomach biopsy. Within the same region, the proteomes were similar as evidenced by the large Spearman correlation coefficients (≥ 0.7) between the samples. The 58 cancer sample pairs were collected from patients diagnosed with late stage gastric cancer.
Data exclusions	No data were excluded from the analyses.
Replication	All attempts at replication were successful.
Randomization	All samples were collected from 7 regions of the stomach based on the anatomy.
Blinding	The investigators were not blinded to group allocation during data collection and analysis. The purpose of the manuscript is to investigate the gene expression profiles of mucosa from 7 anatomic regions of the stomach.

Reporting for specific materials, systems and methods

Materials & experimental systems

n/a	Involved in the study
☒	☐ Unique biological materials
☒	☐ Antibodies
☒	☐ Eukaryotic cell lines
☒	☐ Palaeontology
☒	☐ Animals and other organisms
☐	☒ Human research participants

Methods

n/a	Involved in the study
☒	☐ ChIP-seq
☒	☐ Flow cytometry
☒	☐ MRI-based neuroimaging

Human research participants

Policy information about studies involving human research participants

Population characteristics	The stomach mucosa samples were obtained from Affiliated Hospital Cancer Center, Academy of Military Medical Sciences, Beijing.
Recruitment	All participants were recruited randomly.
